# Costal cartilage mimics urate on DECT

**DOI:** 10.1007/s00256-025-04980-9

**Published:** 2025-07-04

**Authors:** Anthony J. Doyle, Michel K. Nieuwoudt, Jason Woon, Hannah M. Matthews, Nicola Dalbeth

**Affiliations:** 1 Anatomy and Medical Imaging, Faculty of Medical and Health Sciences, University of Auckland, 85 Park Rd Grafton, Auckland, 1023 New Zealand; 2 Science Centre, University of Auckland, 302 - Bldg 302, 23 Symonds St, Auckland, 1010 New Zealand; 3 Department of Medicine, Faculty of Medical and Health Sciences, University of Auckland, Room 502-201D, 85 Park Rd Grafton, Auckland, 1023 New Zealand; 4 Radiology Department, Auckland City Hospital, Te Toka Tumai, 2 Park Rd Grafton, Auckland, 1023 New Zealand

**Keywords:** Urate, Dual-energy CT, Gout, Costal cartilage, Artefact, Mimic

## Abstract

**Objective:**

To establish whether the material in costal cartilages isoattenuating to urate on dual-energy CT is, in fact, urate.

**Materials and methods:**

Under the Human Tissue Act, costal cartilage specimens from 12 donated embalmed cadavers were examined with DECT before and after storage in air, ethanol, and saline. Cadaveric tophi were examined as positive controls. The storage fluid was analyzed for urate. Raman spectroscopy was performed. The results were compared using Student *t*-tests and ANOVA.

**Results:**

For cadavers 1–6, initial DECT isoattenuating volumes were 0.3–1.91 cm^3^ (mean 1.1). Three-month delayed volumes for those stored in air were 0.24–1.6 cm^3^ (mean 0.95). For those stored in ethanol, all volumes were zero (*p* = 0.009). For cadavers 7–12, initial volumes were 0.43–1.45 cm^3^ (mean 1.0). Three-month delayed volumes stored in distilled water were 0.13–0.62 cm^3^ (mean 0.3). Thirteen-month delayed volumes were 0–0.39 cm^3^ (mean 0.14, *p* = 0.001). Urate in the storage fluid for the costal cartilages was zero but that in the tophus storage fluid was high (1002 and 338 umol/L). The absent 632 cm^−1^ Raman spectra peak of costal cartilages 1 and 2 confirmed that no urate was present.

**Conclusion:**

The isoattenuating material in costal cartilages on DECT is not urate, but other material that simulates the attenuation profile of urate under certain conditions. In clinical practice, isoattenuating material in costal cartilages on DECT can be regarded as artifactual.

## Introduction

Dual-energy CT (DECT) is widely used in both the clinical management of gout and in research, based on the ability of the technique to identify (and quantify) deposits of monosodium urate crystals (MSU). That identification revolves around the attenuation profile of urate at two different X-ray energy levels (tissue decomposition). Previous publications have suggested the presence of MSU crystals in body tissues other than joints and tendons, based on DECT characteristics, including in costal cartilage [1–3].

Various mimics of urate have been described, however [4], and it is of clinical significance to verify which materials identified on DECT are actually urate and which are not. This study aimed to clarify the nature of urate-like material shown in costal cartilages on DECT and, in particular, to establish whether the material in costal cartilages with similar characteristics to urate on dual-energy CT is, in fact, urate.

The null hypothesis of this study was that the material simulating urate within costal cartilages on DECT would behave in an identical manner to urate using DECT and other means of analysis.

## Methods

### Human tissue

In a prospective manner, costal cartilages were harvested from 12 embalmed donated cadavers. None had a documented history of gout. Equal numbers of males and females were included. The average age was 73 years (range, 47–91 years). Two tophi (one larger tophus from an elbow, the other smaller tophus from a great toe) from embalmed cadavers with a history of gout were analyzed as positive controls in some experiments. The presence of MSU crystals within the tophi was confirmed by polarizing light microscopy. Collection and use of cadaveric tissue specimens accorded with the national Human Tissue Act 2008. Specific ethics approval and informed consent were not required.

### Storage

The costal cartilage specimens were stored in 100-ml plastic specimen containers under differing conditions.

A first group of 12 specimens (right and left second costal cartilage) were dissected from six cadavers (numbered 1–6). Those from the right side were placed in 50 mL of 70% ethanol added to the containers (the standard solution for storing urate specimens, since urate has low solubility in ethanol [5]) and those from the left were kept in air. The volume of fluid was chosen to completely immerse the specimens while being reproducible and not over-filling the containers. DECT was performed immediately after harvesting. The samples were stored in the same media (ethanol or air), and scans were repeated after a delay of 3 months.

Based on the results from the first set of specimens, a second set of six specimens from six different cadavers (numbered 7–12) was dissected and scanned, first in air and then in 50 mL of distilled water immediately, and then after periods of 3 months, 5 months, and 13 months. The two positive control tophi were also scanned in distilled water along with the costal cartilage specimens from the 3-month timepoints.

### DECT technique

All specimens were scanned on the same twin tube CT scanner (Somatom Flash, Siemens Medical, Erlangen, Germany). The two tubes were operated at 100 kVp and 140 kVp (with tin filtration) respectively, currents of 21 mA and 19 mA respectively, slice thickness 0.75 mm, pitch 0, and field of view 16 cm. Reconstruction was 512 × 512 matrix, 1-mm thickness at 1-mm intervals using a medium soft Br59 kernel. The results were analyzed on a commercial software platform (Syngo Via, Siemens Medical). The “gout” algorithm on this platform codes urate and other material of similar attenuation profile (including the tabletop and some areas of skin and toenails) as green. The term “isoattenuating” will be used in this paper to refer to such material.

For cadavers 1–6, the scans were performed immediately after harvesting and at 3 months. For cadavers 7–12, the scans were performed immediately and at 3, 5, and 13 months. For the tophi, the scans were performed immediately and at 2 and 10 months. The time spacing between scans was based partly on scanner availability and partly on the knowledge that urate has very low solubility in water and ethanol and, therefore, would not be expected to change over a shorter period [6].

### Histological and Raman spectroscopic analysis

Attempts were made to verify the presence or absence of urate in the costal cartilage specimens by anatomical pathology using a previously described microscopic technique [7]. This was not successful because of the friable nature of the specimens that prevented effective sectioning, despite lengthy decalcification.

An attempt was also made to identify urate in the specimens using Raman spectroscopy, which provides a non-invasive and rapid method that can identify the presence of monosodium urate crystals by a characteristic peak for MSU at 632 cm^−1 ^[8]. Raman spectra of specimens 1 and 2 were recorded with an EmVision CT Raman spectrometer with fiber optic probe, using 830-nm excitation at 35 mW. The spectra were acquired by touching the 2-mm diameter probe tip on the selected region of the specimen; collection time was 10 s.

### Fluid analysis

The fluid in which the specimens were kept was then analyzed for urate. Samples were analyzed from the specimens from cadavers 1–6 that were kept in ethanol, all six of cadavers 7–12 kept in distilled water, and the two tophi. Urate concentrations were measured on a Hitachi c311 autoanalyzer (Hitachi High Technologies Corporation, Tokyo, Japan) by enzymatic colorimetric assay (Roche, Mannheim, Germany).

### Statistical analysis

Means and standard deviations of the DECT results were calculated and compared using paired Student *t*-tests and repeated measures analysis of variance (ANOVA) (GraphPad Prism).

## Results

### DECT urate values of costal cartilages stored in air or ethanol for 3 months

**Fig. 1:**
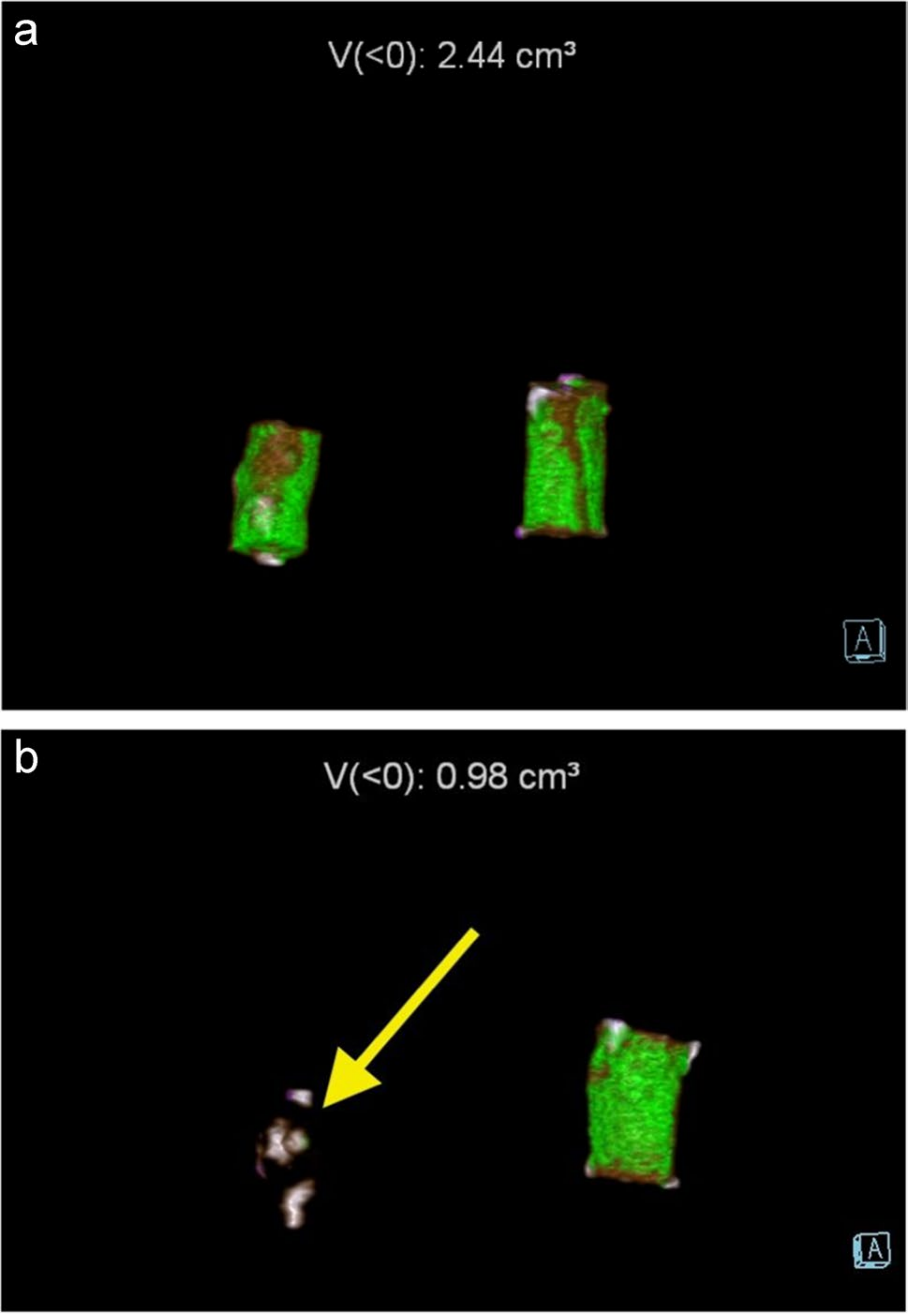
Examples of costal cartilage scans immediately after harvesting and 3 months after storage in ethanol or air. **a** Immediate scan of left and right costal cartilages stored in 70% ethanol and air respectively shows isoattenuating material in both, with total volume 2.44 cm^3^. **b** Scan of same specimens after 3 months—left costal cartilage stored in 70% ethanol (yellow arrow) shows complete disappearance of isoattenuating material; right costal cartilage stored in air is stable with isoattenuating material 0.98 cm^3^

DECT isoattenuating volumes for costal cartilages from cadavers 1–6 (6 in air and 6 in ethanol) are shown in Table 1. For cadavers 1–6, the initial DECT obtained immediately after harvesting showed a range of isoattenuating volumes for the specimens stored in air of 0.30 to 1.91 cm^3^ (mean 1.10 cm^3^). The 3-month delayed scans showed a range of isoattenuating volumes from the specimens stored in air of 0.24–1.60 cm^3^ (mean 0.95 cm^3^), and this was not significantly different from volumes at the initial scan. For those stored in ethanol, the range of initial volumes was 0.08–1.59 cm^3^ (mean 0.94 cm^3^). The 3-month delayed volumes for the specimens stored in ethanol were all 0 cm^3^ (compared with initial volumes, *P* = 0.009). Figure 1 shows examples of scans immediately after harvesting and 3 months after storage in air or ethanol.

**Table 1:** DECT isoattenuating volumes (cm^3^) for 12 costal cartilage specimens from cadavers 1–6 (6 in air and 6 in ethanol)

Cadaver number	Air initial	Air delayed 3 months	Ethanol initial	Ethanol delayed 3 months
1	0.3	0.24	0.08	0
2	1.01	0.94	1.43	0
3	0.88	0.78	0.61	0
4	1.36	0.98	1.08	0
5	1.14	1.13	0.86	0
6	1.91	1.6	1.59	0
**Mean (SD)**	1.10 (0.53)	0.95 (0.45)	0.95 (0.55)	0 (0)

### DECT urate values of costal cartilages stored in distilled water over 13 months

**Fig. 2:**
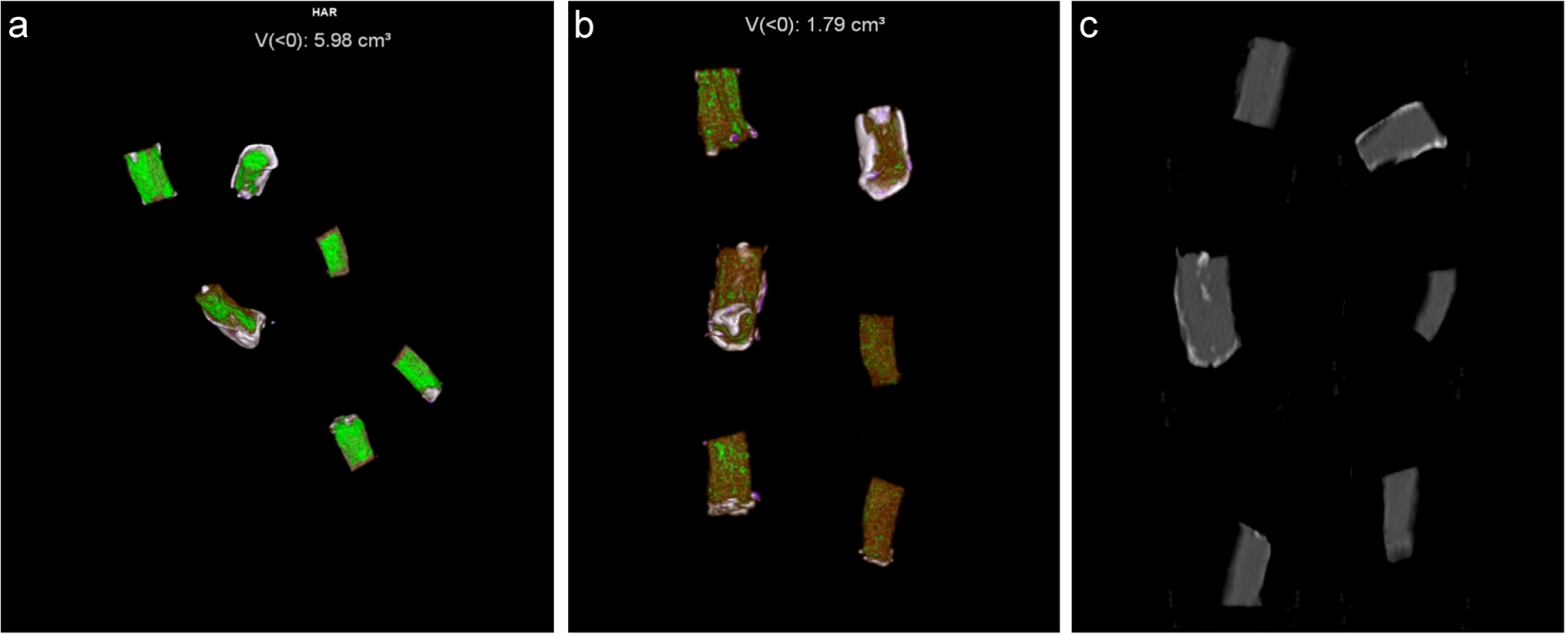
Serial DECT isoattenuating volumes of costal cartilage from cadavers 7–12 during storage in distilled water. **a** Initial scan of six costal cartilage specimens shows isoattenuating material of total volume 5.96 cm^3^ (mean 1.0 cm^3^). **b** Delayed scan after 3 months in distilled water shows pronounced drop in isoattenuating volume to total 1.79 cm^3^ (mean 0.3 cm^3^). **c** Bone window MPR of costal cartilages showing small (mostly peripheral) areas of calcification in some specimens, not corresponding closely to isoattenuating material

**Fig. 3:**
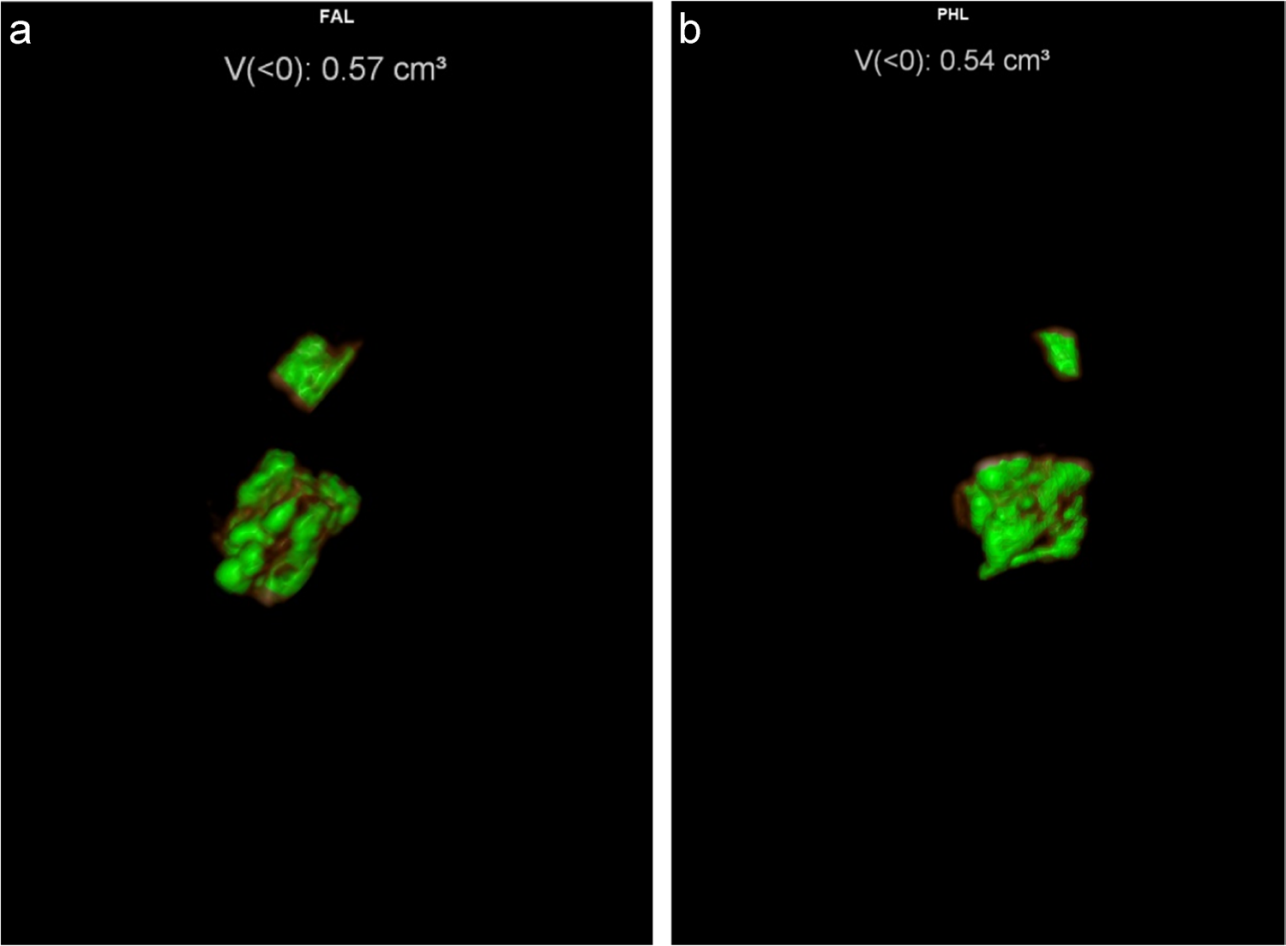
Serial DECT isoattenuating volumes of tophi during storage in distilled water. **a** Initial scan of two tophi shows isoattenuating material of total volume 0.57cm^3^. **b** Delayed scan after 2 months in distilled water shows minimal drop in isoattenuating material to 0.54 cm^3^

DECT isoattenuating volumes for costal cartilages from cadavers 7–12 are shown in Table 2. For cadavers 7–12 (all specimens stored in distilled water), the initial scans showed a range of isoattenuating volumes of 0.43–1.45 cm^3^ (mean 1.00 cm^3^). Over 13 months, these progressively declined to 0.00–0.14 (mean 0.39 cm^3^), ANOVA *P* = 0.001. Figure 2 shows examples of scans immediately after harvesting and 3 months after storage in distilled water, as well as a bone window MPR image showing the degree and location of mineralization in the specimens. The positive control tophi showed initial urate volumes of 0.5 and 0.08 cm^3^, declining very slightly over 2 months to 0.47 and 0.07 cm^3^ respectively (Fig. 3). After 10 months’ storage, the volumes were 0.42 and 0.07 cm^3^, respectively.

**Table 2:** Serial DECT isoattenuating volumes of costal cartilage specimens from cadavers 7–12 during storage in distilled water

Time	Initial	3 months	5 months	13 months
	Volume cm^3^	Volume cm^3^	Volume cm^3^	Volume cm^3^
**Costal cartilage** ** Cadaver number**				
7	0.96	0.13	0.18	0.14
8	1.45	0.62	0.31	0.39
9	0.79	0.16	0.09	0.05
10	0.43	0.16	0	0
11	1.32	0.36	0.15	0.15
12	1.02	0.36	0.09	0.08
**Mean (SD) costal cartilage**	1.00 (0.37)	0.30 (0.19)	0.14 (0.11)	0.14 (0.14)

### Storage fluid urate concentrations

Storage fluid urate concentrations are shown in Table 3. Biochemical analysis showed urate concentrations of 0 µmol/L in all of the costal cartilage fluid storage specimens (both ethanol and distilled water). In contrast, the distilled water in which the two positive control tophus samples were stored had urate concentrations of 1002 and 338 umol/L.

**Table 3:** Storage fluid urate concentrations

	Urate concentration in fluid µmol/L
**Costal cartilage** **Cadaver number**	
**Time**	**24 months**
Cadaver 1 in ethanol	0
Cadaver 2 in ethanol	0
Cadaver 3 in ethanol	0
Cadaver 4 in ethanol	0
Cadaver 5 in ethanol	0
Cadaver 6 in ethanol	0
**Time**	**16 months**
Cadaver 7 in distilled water	0
Cadaver 8 in distilled water	0
Cadaver 9 in distilled water	0
Cadaver 10 in distilled water	0
Cadaver 11 in distilled water	0
Cadaver 12 in distilled water	0
**Tophus**	
**Time**	**13 months**
Tophus 1 in distilled water	338
Tophus 2 in distilled water	1002

### Raman spectroscopy

**Fig. 4:**
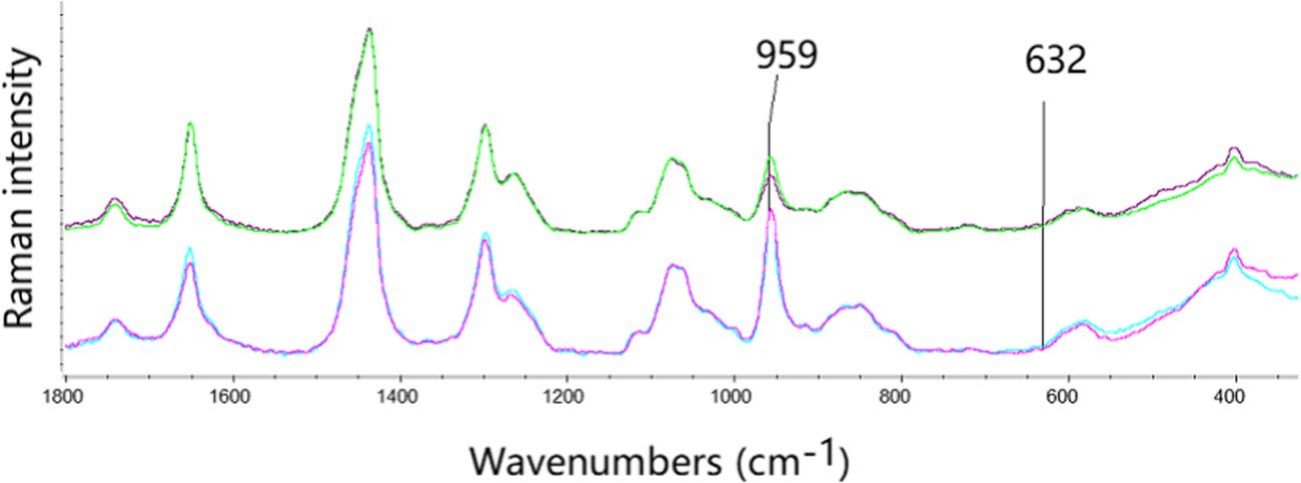
Comparison of Raman spectra from costal cartilages 1 and 2, measured at 830 nm, showing peaks at 959 cm^−1^ for collagen and calcium carbonate but no urate peak at 632 cm^−^^1^

The Raman spectra measured of the two specimens 1 and 2 are shown in Fig. 4. While Raman peaks characteristic for collagen and calcium carbonate at 959 cm^−1^ were present, no characteristic monosodium urate peak at 632 cm^−1^ was detected (Fig. 4), confirming that no MSU was present.

## Discussion

The key finding in this study is that isoattenuating material shown by DECT in costal cartilage specimens behaved quite differently from urate in control tophi in terms of both changes in DECT values and on chemical analysis of fluid in which the specimens were stored. The isoattenuating material in costal cartilages rapidly diminished on storage in either ethanol or distilled water, while persisting in air storage, whereas the proven urate in tophi, by contrast, was stable over a long period. It has been previously shown that urate is almost completely insoluble in ethanol [5], indicating that the disappearance of isoattenuating material in ethanol storage cannot be attributed to leaching of urate out of the tissue. Analysis of the storage fluid for the costal cartilages over periods of up to 2 years showed no detectable urate, while substantial levels of urate were detected in distilled water in which the tophi had been stored. The latter indicates that, despite the low solubility of urate in water [5, 9], some urate from the tophi did dissolve in the storage fluid. Were there urate in the costal cartilages, it would be expected that urate would be detectable in that storage fluid also.

From the results above, it is clear that the isoattenuating material in costal cartilage does not behave in the same manner as urate, either on DECT under differing storage conditions or on chemical analysis of the fluid in which the samples were stored. There is no evidence, in this series of experiments, of any urate in the costal cartilages. Therefore, the isoattenuating material seen in costal cartilage on DECT is not urate but a substance or combination of substances with a similar attenuation profile to urate on standard DECT before being placed in liquid storage media.

The only requirement for a substance to mimic urate on DECT is that it have a similar attenuation profile at the X-ray tube energy levels used in the scan. In this study (as with most clinical DECT), two X-ray energies are used (80 and 140 keV, the latter with a tin filter as recommended and verified as giving results with least artifact [10]). The attenuation profile of materials at these energies depends on the electron density of the material (by Compton scattering, which increases with increasing X-ray energy) and the atomic number of the chemical components of the material (by the photoelectric effect, which decreases with the third power of the X-ray energy). Therefore, on theoretical grounds, it is perfectly possible for a substance that is not urate to have a similar attenuation profile, provided that it has similar electron density and contains at least some similar atoms to cause similar photoelectric effect attenuation.

The isoattenuation of costal cartilages disappeared altogether on storage in ethanol and progressively decreased over time in distilled water. Such storage would not change the atomic number of the constituent molecules but could change the physical density (and thereby the electron density) of the costal cartilage, depending on how much fluid it absorbed. This, presumably, alters the attenuation profile sufficiently that the tissue decomposition algorithm no longer identifies the costal cartilage as containing urate. It would also explain why the drop in isoattenuating volume over time differs between storage in ethanol from storage in water, if costal cartilage absorbs the two liquids at different rates.

Studies of the chemical composition of costal cartilage are somewhat limited but one recent study showed significant variation in the proportions of chondrocytes, collagen, and mineralized tissue present in a series of 25 specimens [11]. Another recent study showed relatively high levels of glycosaminoglycans, mineralization, and chondrocytes compared with articular cartilage but overall lower levels of collagen [12]. This chemical heterogeneity likely contributes to the presence of isoattenuating material on DECT despite the evident lack of urate present. Another recent biophysics study has highlighted the challenges of analyzing more than three chemical elements using standard DECT techniques [13].

Although mineralized tissue (especially that containing calcium) can produce artifactual isoattenuation with urate on DECT [14], in the current study, the fairly dense calcified material did not produce persistent isoattenuation after storage in fluid (Figs. 1 and 2). As shown in Fig. 2c, only two of six specimens from cadavers 7–12 had significant calcification and most of this was peripheral, unrelated to the prominent isoattenuating material. Therefore, artifactual isoattenuation in costal cartilage in this study cannot be attributed to calcium deposition.

Several emerging CT and radiographic methods using photon-counting detectors have shown promise for improving both the spatial and chemical resolution of imaging in crystal arthropathies (and other conditions). In particular, it may be possible in the near future to reliably discriminate between tophi, calcium pyrophosphate, and hydroxyapatite and to detect and characterize very small deposits of urate. These methods, however, remain at a pre-clinical stage where they can produce images of tissue samples or small body parts only [15–18].

The strengths of this study include the use of both DECT and chemical analysis to establish that the isoattenuating material in costal cartilages does not behave as urate, the uniformity of results from multiple cadavers, use of positive control tophi, and the examination of the specimens over a prolonged period of time. The volumes of isoattenuating material are automatically generated by SyngoVia, the software platform used for DECT analysis. This particular study did not require any manual segmentation, measuring, or interpretation. In that context, observer bias and agreement do not come into play.

The limitations include the inability to accurately measure the density of the costal cartilages (and to thereby establish that density changes account for the variable attenuation on DECT) and the fact that we have not been able to clarify which compound(s) account for the isoattenuating behavior.

This work establishes that isoattenuating material within costal cartilages on DECT is not urate. Similar considerations should be kept in mind when examining other body structures in which the presence of urate correlating with isoattenuation has not been confirmed by direct tissue analysis. Recently, our group demonstrated that, contrary to some other investigations, urate deposition is not present in major vascular structures in patients with gout [19]. Although it is probably reasonable to assume that isoattenuating material at well-studied anatomic sites in people with gout (or those with elevated urate levels) is in fact MSU, the same assumption cannot be made for isoattenuating material in other locations.

## Conclusion

The material in costal cartilages previously thought to represent urate on DECT is not urate, but other material that simulates the attenuation profile of urate under certain conditions.

In clinical practice, material in costal cartilages showing a similar profile (isoattenuating) to urate on DECT can be regarded as artifactual.

## Data Availability

All data in this study is available for review on request and has not been fabricated.
